# Echoes of the Month

**Published:** 1923-12

**Authors:** 


### ECHOES OF THE MONTH.

### DEAN OF ST. PAUL'S ON NATIONAL DECAY.

Speaking on "National Decay and Regeneration" at Edinburgh, the Dean of St. Paul's said that only one kind of natural selection was favoured by modern life—that against disease. That had gone on so rapidly that most of the epidemics which formerly ravaged the cities had assumed a much milder type, and were far less destructive to life. To introduce a savage into any European town was to sentence him to certain death. The modern man might be physically a poor creature, but he had developed a new power of resisting microbes. It was the unexampled decline in the death-rate, due partly to sanitation and medical skill, but partly also to increased adaptation to town life, which had created the very menacing problem of over-population, to which the most bigoted could no longer shut their eyes, with a million unemployed to feed out of the taxes. The decline in the birth-rate from 36 in 1878 to 20 in 1922 had prevented a social revolution, and the death of many millions by famine, but it had not gone far enough. The decline in the rate was itself dangerous, because it was steadily impairing the quality of the population. They were breeding from the bottom and dying off at the top. In each generation the cream of all classes was skimmed off, raised to a better social position, and there sterilised. Among the finest strata of the population, physically, intellectually, and morally, were the members of the learned professions, and it was therefore a bad symptom that at present the lowest birth rates were those of the doctors, ministers of religion and teachers. And if it was the skilled workman who was filling the cradle there would be much less ground for alarm, but it was the slum dweller, the sub man, the untaxed dole receiver who was the father of the next generation, or by far more than his share of it. The highest birth rate of all was that of the feeble minded.

### DRAIN DEFECTS NOT DANGEROUS.

According to Dr. Wynne, M.O.H. for Sheffield, there is no discoverable danger to health in an escape of gas from a drain or sewer. Dr. Wynne recalls the fact that Professor Delépine, of Manchester, found no difference in the health of animals kept in a room in which the air was entirely derived from a sewer and in those kept in a room in which the air was drawn from an adjoining garden. And he instances experiments made in Leeds which showed in the case of several thousand houses that those in which drain defects were present had no worse record of typhus or diphtheria than the others.

### NON-OPERATIVE TREATMENT OF CANCER.

*Truth* states that the Board of Management of the Battersea General Hospital have resolved to reopen their closed cancer wards. "An opportunity is thus offered of conducting a definite test of the value of non-operative treatment of cancer, with the probability that, if any substantial number of satisfactory results are obtained, useful light will be thrown upon the causes of the disease, which have hitherto been sought in vain by different and much more costly methods. The cancer wards at Battersea, containing fourteen beds, are in every way suited for the reception of patients; they are equipped with every requirement for their treatment, both by dietetic methods and every known scientific process for fighting the disease, except the surgeon's knife; and the patients will be in the hands of a medical staff, under Dr. Robert Bell, who are all specialists in non-operative treatment of cancer."

### CHILD PSYCHOLOGY.

Speaking on "Children of School Age" at Carnegie House, Dr. Niall said that the three great forces governing every human being from birth onwards are heredity, environment and training. Whatever developments await us in later life, wherever we go and whatever we do, we cannot altogether escape or outlive these primary factors. Heredity is a fixed and immutable condition and acts both physically and mentally. But environment and training are equally important in the ultimate effect upon the making or marring of the potential citizen. A certain amount of physical freedom, encouragement to romp and play games, should be given to every child, while simple but artistic colour schemes, flowers to look at and plenty of air and sunshine, help to form sturdy limbs and a cheerful outlook. Dr. Niall mentioned the effect that neglected adenoids have upon the children who live in the suburbs where houses stand in monotonous streets, each one exactly like the next. The front parlour is stuffy and unused, and the children—not being allowed to play in the streets upon the score of "respectability"—develop into stupid, lethargic little beings simply from lack of air, exercise and imagination.

### ONE LEG LARGER THAN THE OTHER.

Girls who drive their own cars are warned by a Paris doctor that they are in danger of having one leg grow larger than the other. This is the limb that presses the accelerator. The physician declares that several cases of bunchy muscles in the calf of the right leg have been called to his attention. He also declares that too much tennis is having an influence on the younger generation of French girls, making them "right-sided," with a muscular development that throws their bodies out of the proportion and symmetry needed for the highest beauty.

### SUNLIGHT TREATMENT IN LONDON.

At the suggestion of Sir Henry Gauvain an experiment was conducted at Stowey House open-air school, Clapham Common, during the summer months to ascertain how far sunlight treatment could be utilised in day open-air schools. Thirty-five boys were selected, with their parents' consent, by the school medical officer. The headmaster reports that during the first week the lads worked at their class and manual lessons in light shirts, short pants, socks and shoes. The second week shirts were discarded, and so on during succeeding weeks, until they wore nothing more than their breeches. Their bodies were browned without signs of redness, a factor essential to correct treatment. The headmaster reports that as a result of a few weeks' treatment the boys appeared more alert, more energetic, and particularly happy under the new conditions. On their return to school after the summer holidays it was necessary to acclimatise them again, but this time for only one or two days were they compelled to wear shirts whilst at work. The school medical officer reports that the results of the treatment were sufficiently promising to make it highly desirable for the experiment to be continued next summer, and the Education Committee has decided that this should be done.

### PLASTIC SURGERY AT DOLLIS HILL.

An international clinic of plastic surgery is to be established at St. Andrew's Hospital, Dollis Hill, where patients of the professional and middle classes can now be treated. The operations are extremely delicate and often very prolonged. Three or four hours is not unusual, and sometimes an operation which begins at 1 p.m. does not finish until 8 o'clock at night. A remarkable case was that of a nurse who was so badly burned that her jaw was drawn down, by contraction of the skin, to her chest. Major Gillies, the plastic surgeon, grafted skin from her abdomen on her arm; when the skin had taken root on the arm he severed it from the abdomen, raised the arm to the neck, and grafted the skin there. The nurse is back at work.

### THE NEED OF RAT CLUBS.

A strong case exists for the formation of "Rat Clubs" in connection with every industrial undertaking which suffers from these creatures [says the Trade and Engineering Supplement of the *Times*]. The object of these clubs, of which a few exist already, is primarily, of course, hygienic. It has been found, however, that considerable enthusiasm for the "hunt" itself is soon awakened among the members. Thus there is encouraged the breeding of terriers which are good "ratters," and also the cultivation of greater skill in attacking and destroying the pests. In this way the rat population of a district or factory is gradually worn down until it becomes so small as to be easily extinguished altogether. The work of the rat club ought to be an object of interest to the employer, who stands to benefit very considerably by it. Every reasonable opportunity for organizing hunts and carrying them out should be afforded. In addition the employer ought to co-operate with the club secretary in consolidating the ground won. The sewers and drainage pipes of most workplaces are extremely defective in this respect. They make it, indeed, almost impossible to prevent the immigration of fresh "blood" into places which have been cleared.

### TESTING DIPHTHERIA IN EDINBURGH.

Dr. William Robertson, M.O.H. of Edinburgh, has begun a crusade of prevention against diphtheria among school children by means of the Schick test. This method enables the doctor to discover those susceptible to the disease. There are one or two striking features regarding the susceptibility of the population which are worth noticing. Among newborn infants only 7 per cent. are susceptible, but in the first year of life the susceptibility rose to 43 per cent., and between the ages of two and five it increased to 63 per cent. When adult age is reached the susceptibility fell to 10 per cent. Between 80 and 90 per cent. of the infants born could resist attack because they came into the world with a toxin circulating in their blood stream. As the infants grow older the toxin gradually disappeared.

### TALLER WOMEN.

A New York lady doctor who has been measuring women in American Universities for thirty years has come to the conclusion that they are growing taller. Dr. Mosher holds that the increase in height of the average woman is not due to more food or better food, but to more exercise, sport and work, and, incidentally, to the wearing of fewer and more sensible clothes. The average woman measured in 1921-22 was 1.2 inches taller than the average woman measured in 1891-2. Similar measurements of women made at Vassar College in the same years show an increase of 1.5 inches in the average height. Dr. Moshler finds that not only is the average woman taller, but she has a larger waist and her bodily proportions are better from the standpoint of health.

### FOREHEADS AND BRAINS.

Speaking on "The Brain and Intelligence" at the Ethnological Society, Dr. Bernard Hollander said that if we took the lowest animal through the different species until we reached the highest apes, we should find that corresponding to the growth of intelligence the frontal lobes had increased in size. Moreover, the expansion of the forehead in men engaged in intellectual pursuits had been repeatedly demonstrated by actual measurements. The examination of prehistoric skulls had revealed that the progress of civilisation had resulted in raising the anterior and flattening the posterior parts of the head. But the forehead might be large, lofty, and vaulted without unusual intelligence. The quality of the brain structure, the state of its blood supply and nutrition, the conditions of the bodily organs all influenced mental energy. If the necessary character stimulus was lacking the person might be a mer dreamer.

### SAVED BY THE PNEUMONIA BLOUSE.

Probably the greatest sanitary reform of our day (says a writer in the *Manchester Guardian*) was the introduction, shortly after the close of the Victorian era, of what was known at the time as the "pneumonia blouse." All the "health weeks" and all the "propaganda" about health with which we have since become rather painfully familiar could not have done it. But the dressmaker did it, as it were, with a wink of an eyelid. About the same time, or a little later, men discovered one loophole left for ventilation and added spats to their attire. Women have certainly become healthier since they discarded most of the unnecessary garments in which their grandmothers swaddled themselves. But, I am always asked, or was when I dared to propound these views in person, why are coughs and colds and all the rest of it so much more prevalent in winter? The answer is very obvious. It is because in winter we live indoors and keep our windows shut. It is in winter that we pile on clothes and paralyse our heat-regulating mechanism. Above all, it is in winter that we congregate. We assemble and meet together in the church, in the theatre, at political and other meetings; we close the windows in our railway carriage and our tramcar. We are no longer sweating over lawn tennis and healthily cooling ourselves by lying full-length on the damp grass.

### EDUCATION IN HYGIENE.

"Here in Holland," writes the medical correspondent of the *Observer*, from Utrecht, "even more than in Great Britain, I find the medical students slaving away at the details of anatomy and the dosage and preparation of the vast rubbish heap of nostrums which the Materia Medica has inherited from the Middle Ages, whilst the simple laws of personal health, of ventilation and diet, and exercise and clothing and nose-breathing, and so forth, are adequately taught neither to medical students nor by medical men to the public. The personal application of the laws of hygiene is our urgent need. None of us desire our own destruction by disease, whether of life, or of the joy of life; no suicide elects to die of pulmonary tuberculosis or of Bright's disease: but millions do those things for which, in due season, they will thus die. No one teaches them; so far as I can discover, no one teaches them in Holland, and nothing in the medical curriculum promises that the doctors of the next few decades will undertake the task. Immense is the contrast with the United States and Canada, where the Press is the medium of public education, persistent and well-devised.

### REJUVENATING WOMEN.

Dr. Jaworski, of Paris, claims to have discovered a means of rejuvenating women by means of the transfusion of a few drops of blood of a particularly vigorous young person into the veins of an elderly one. Dr. Jaworski began experimenting on animals, and great success attended his efforts, for by the introduction of younger blood he converted a failing she-goat of thirteen years into a frisky, playful creature, brought back new bark and bite to a lady-dog whose career had seemed to be approaching its end after fourteen years, and opened out a fresh, alluring vista of palpitating life to an old mare of twenty-four summers. Having satisfied himself that the theories which he has nursed for a long time are well founded—a belief in which he is supported by eminent colleagues—Dr. Jaworski has recently been endeavouring to ward off old age from women patients, and he has, he says, obtained most positive and convincing results, which he proposes shortly to report officially to the faculty. One of the difficulties that confronts him in his desire to do this is the natural objection of the ladies con cerned to associate themselves with affirmation of the success of the experiments.

### MEDICINE AND LITERATURE.

Delivering the first David Lloyd Roberts lecture to the Royal College of Physicians on "Personal Relations between Medicine and Literature," Mr. Edmund Gosse said that men of letters and medical men had for centuries betrayed an identity of sympathy not to be found between doctors and men of any other profession. The barrier between imaginative literature and medical science was broken down by the publication in the last year of the seventeenth century of Sir Samuel Garth's poem, "The Dispensary." He communicated on terms of perfect equality with the most eminent wits in the coffee-houses—a new thing then—and the close relation between literature and medicine was completed in that year when Garth secured the body of the poet Dryden and brought it to the Royal College of Physicians, then in Warwick Lane, from which a great procession left for Westminster Abbey. Garth pronounced a Latin oration first and in the following nineteen years of his life the good relations between literature and medicine became the rule in cultivated circles rather than the exception. The physician was welcome at the wit-combats of the clubs and coffee-houses, where he introduced an element of humanity and an experience of suffering and patience which were not lost on his more volatile companions. Later, Dr. John Arbuthnot, the friend and adviser of Pope and Swift, took up the mantle of Garth and sat in the circle of wits as "our best-natured man." For a quarter of a century he was a planet in the constellation of wits, to which he added a lustre of his own.

